# COVID-19 and MIS-C treatment in children—results from an international survey

**DOI:** 10.1007/s00431-023-05179-7

**Published:** 2023-09-06

**Authors:** Daniele Donà, Chiara Minotti, Tiziana Masini, Martina Penazzato, Marieke M. Van Der Zalm, Ali Judd, Carlo Giaquinto, Marc Lallemant, Survey Respondents Consortium, Antonia H. M. Bouts, Eric McCollum, Alasdair Bamford, Pablo Rojo, Alfredo Tagarro, Nanny Nan P., Eduardo Lopez, Sonia Bianchini, Giangiacomo Nicolini, Alla Volokha, Luca Pierantoni, Stefania Bernardi, Vania Giacomet, Tinsae Alemayehu, Kanokkron Swasdichai, Elio Castagnola, Charl Verwey, Petar Velikov, Paolo Palma, Fatima Mir, Rhian Isaac, Timo Jahnukainen, Cristina Calvo, Nicolaus Schwerk, Omotakin Omolokun, Agnese Tamborino, Marinella Della Negra, Shubhada Hooli, Gary Reubenson, Mazimpaka A., Devika Dixit, Qalab Abbas, Taryn Gray, Marta Gonzalez Vicent, Kate Webb, Grace Damasy, Andrew Riordan, Maria Francelina Lopes, Suparat Kanjanavanit, Steven Welch, Andrea Lo Vecchio, Silvia Garazzino, Helen Payne, Suchada Ruenglerdpong, Katja Masjosthusmann, Malte Kohns Vasconcelos, David Burgner, Davide Meneghesso, Alessandra Meneghel, Elizabeth Whittaker, Joseph Aluoch, Vannee Thirapattarapong, Magdalena Maria Marczyńska, Winnie August, Helena Rabie, Andreas Groll, Guido Castelli Gattinara, Alvaro Madrid, Marial Hierro, Dominique Debray, Shelina Jamal, Elisabetta Calore, Mara Cananzi, Marica De Pieri, Martin Eduardo Brizuela, Chawanzi Kachikoti, George Akabwai, Selam Seged, Tom Wolfs, Christos Karatzios, Marco A. Tovar, Polynary A., Edward Kabeja

**Affiliations:** 1 Department of Women’s and Children’s Health, University of Padua, Padua, Italy; 2 Division of Pediatric Infectious Diseases, Department of Women’s and Children’s Health, Padova University - Hospital, Padova, Italy; 3 WHO Research for Health Department, World Health Organization, Geneva, Switzerland; 4 Desmond Tutu TB Centre, Department of Pediatrics and Child Health, Faculty of Medicine and Health Sciences, Stellenbosch University, Cape Town, South Africa; 5 MRC Clinical Trials Unit at UCL, University College London, London, UK; 6 Faculty of Associated Medical Sciences, Chiang Mai University, Chiang Mai, Thailand

**Keywords:** Off-label, Antivirals, Monoclonal antibodies, COVID-19, Children, Global access

## Abstract

Children have been mostly excluded from COVID-19 clinical trials, and, as a result, most medicines approved for COVID-19 have no pediatric indication. In addition, access to COVID-19 therapeutics remains limited. Collecting physicians’ experiences with off-label use of therapeutics is important to inform global prioritization processes and better target pediatric research and development. A standardized questionnaire was designed to explore the use of therapeutics used to treat COVID-19 and multisystem inflammatory syndrome in children (MIS-C) in pediatric patients globally. Seventy-three physicians from 29 countries participated. For COVID-19, steroids were used by 75.6% of respondents; remdesivir and monoclonal antibodies were prescribed by 48.6% and 27.1% of respondents, respectively. For MIS-C, steroids were prescribed by 79.1% of respondents and intravenous immunoglobulins by 69.6%. The use of these products depended on their pediatric approval and the limited availability of antivirals and most monoclonal antibodies in Africa, South America, Southeast Asia, and Eastern Europe. Off-label prescription resulted widespread due to the paucity of clinical trials in young children at the time of the survey; though, based on our survey results, it was generally safe and led to clinical benefits.

*Conclusion*: This survey provides a snapshot of current practice for treating pediatric COVID-19 worldwide, informing global prioritization efforts to better target pediatric research and development for COVID-19 therapeutics. Off-label use of such medicines is widespread for the paucity of clinical trials under 12 years and 40 kg, though appears to be safe and generally results in clinical benefits, even in young children. However, access to care, including medicine availability, differs widely globally. Clinical development of COVID-19 antivirals and monoclonal antibodies requires acceleration to ensure pediatric indication and allow worldwide availability of therapeutics that will enable more equitable access to COVID-19 treatment.

**Table:** 

**What is Known:** *• Children have been mostly excluded from COVID-19 clinical trials, and, as a result, most medicines approved for COVID-19 have no pediatric indication.* *• Access to care differs widely globally, so because of the diversity of national healthcare systems; the unequal availability of medicines for COVID-19 treatment represents an obstacle to the pediatric population's universal right to health care.*	
**What is New:** *• Off-label COVID-19 drug prescription is widespread due to the lack of clinical trials in children younger than 12 years and weighing less than 40 kg, but relatively safe and generally leading to clinical benefit.* *• The application of the GAP-f framework to COVID-19 medicines is crucial, ensuring widespread access to all safe and effective drugs, enabling the rapid development of age-appropriate formulations, and developing specific access plans (including stability, storage, packaging, and labeling) for distribution in low- and middle-income countries (LMICs). Antivirals and monoclonal antibodies may benefit from the acceleration to reach widespread and equal diffusion.*

**Supplementary Information:**

The online version contains supplementary material available at 10.1007/s00431-023-05179-7.

## Introduction

Although COVID-19 infection is generally mild in children, underlying comorbidities can lead to increased disease severity and higher hospitalization rates, even in pediatric populations [1–3]. Notably, in low- and middle-income countries (LMICs), the impact of COVID-19 in children has been higher [4, 5]. In some areas, access to hospital care and availability of essential equipment might be limited, with consequent higher mortality and morbidity [4, 6].

Besides steroids, most drugs used for COVID-19-specific treatment and multisystem inflammatory syndrome in children (MIS-C) are used off-label [7–9]. Initially, randomized controlled trials (RCTs) for COVID-19 treatment had mainly focused on the adult population and adolescents [8, 10, 11], with the first evidence from pediatric RCT on both MIS-C treatment and monoclonal use for COVID-19 being published only recently in 2023 [12, 13].

In preparation to the Pediatric Drug Optimization (PADO) meeting convened by World Health Organization (WHO) in September 2022 designed to identify priorities for pediatric research and development for COVID-19 treatment, a survey was developed to better understand the availability, extent and type of off-label drugs used for treating COVID-19 and MIS-C in children.

## Materials and methods

In August 2022, a standardized online questionnaire was designed to collect global experiences with different COVID-19 drugs used in children (available in the Supplementary materials). The multiple choice questionnaire was prepared using Google Form ^®^ tools focused on COVID-19 and the other on MIS-C-specific treatments.

The questionnaire was distributed to pediatricians and pediatric infectious disease specialists from PENTA (Pediatric European Network for Treatment of Aids) network, ERN (European Reference Network) transplant child, WSPID (World Society for Pediatric Infectious Diseases), SITIP (Italian Society of Pediatric Infectious Diseases), AfSPID (African Society for Pediatric Infectious Diseases), SASPID (Southern African Society for Pediatric Infectious Diseases), SAPA (South African Pediatric Association), and within networks of collaborators.

### Data collection and analysis

All clinical, demographic, and prescription data were stored in a password-protected, secured server at the University of Padova, Italy. Data were summarized as numbers and percentages (categorical variables).

### Survey drugs

The following categories of medicines were included in the survey, as they are recognized as mainstay treatments for COVID-19 and MIS-C by national and international drug agencies, pediatric infectious diseases experts, and based on a preliminary scoping literature review.

COVID-19 treatments are as follows:

- steroids,
- antivirals (remdesivir, nirmatrelvir + ritonavir, molnupinavir),
- monoclonal antibodies (sotrovimab, casirivimab–imdevimab, regdanvimab, tixagevimab/cilgavimab).

MIS-C treatments are as follows:

- steroids,
- intravenous immunoglobulins (IVIGs),
- acetylsalicylic acid (ASA),
- anakinra

For each category or product, the proposed questions dealt with the reason(s) for use/not use and availability, patient age groups and drug doses, comments on the observed clinical benefits as a result of administration (symptoms resolution and/or non-progression to severe disease), and reported side effects.

## Results

**Fig. 1:**
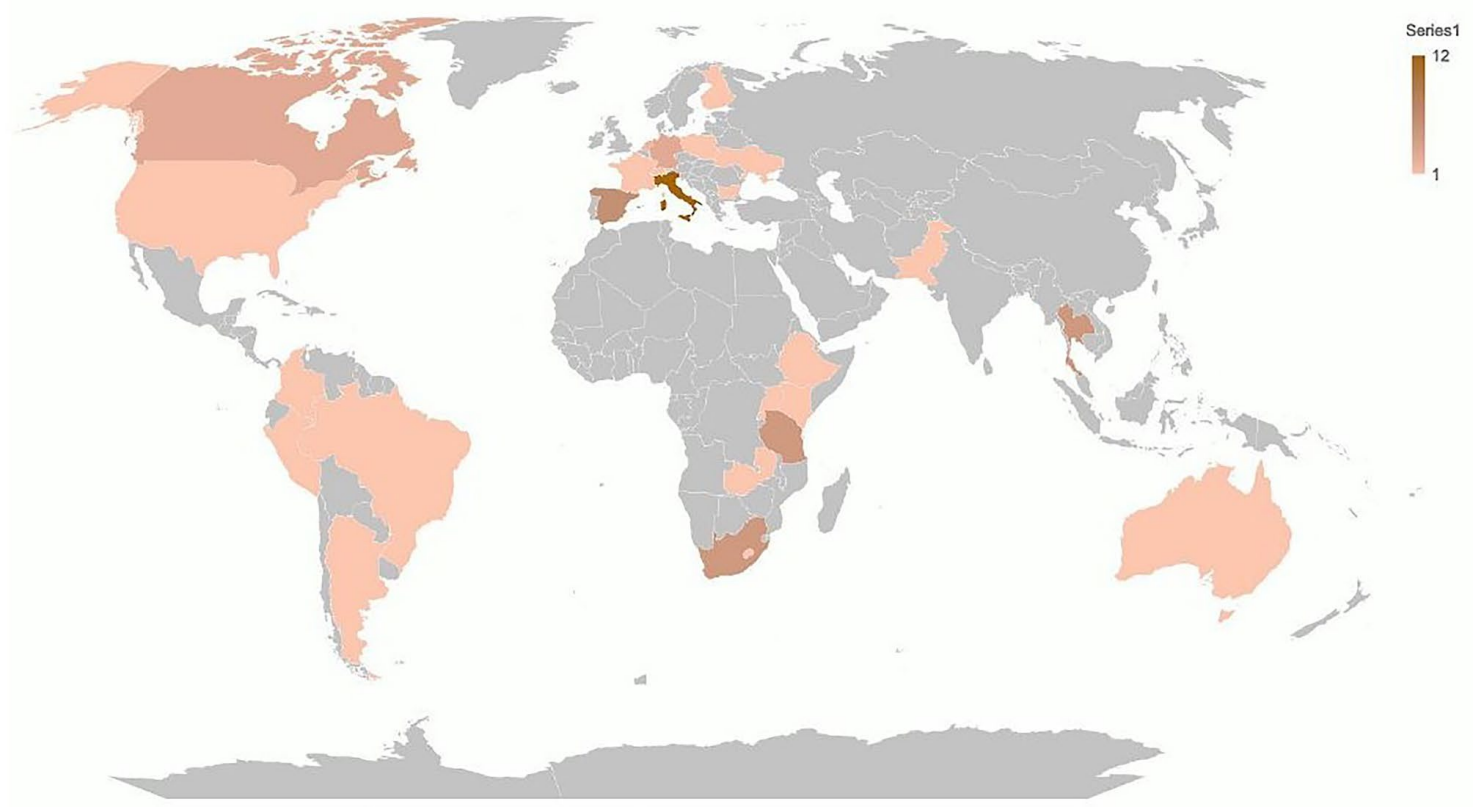
Countries of origin of respondents

The online survey was proposed to 107 practitioners. Seventy-three physicians from 62 sites in 29 countries responded to the survey (Fig. 1), with a maximum of 70 answers (response rate of 65.4%) on single drugs eligible for evaluation. We considered one eligible answer if there were two or more respondents working in the same facility and ward.

Regarding the global availability, steroids were available in most centers, except for one in Tanzania; remdesivir’s availability was also generally widespread; for nirmatrelvir + ritonavir, molnupinavir, and monoclonal antibodies (casirivimab-imdevimab, sotrovimab, tixagevimab-cilgavimab) availability was much more limited. For MIS-C treatment, drugs were reported as available worldwide, except for Tanzania (steroids) and Bulgaria (IVIGs, ASA, and anakinra). Further details are available in the Supplementary materials.

### COVID-19-specific treatment

The use of medications for COVID-19-specific treatment is reported in Table 1.

**Table 1:** Use of medications for COVID-19-specific treatment

	**Yes, % (*n*)**	**No, % (*n*)**	**Medication not available, % (*n*)**	**Medication available but not approved for children with COVID-19, % (*n*)**	**Medication available, not approved for COVID-19, % (*n*)**
**Steroids**	75.6% (53)	21.6% (15)	1.4% (1)	1.4% (1)	0% (0)
**Antivirals**					
**Remdesivir**	48.6% (34)	34.3% (24)	12.9% (9)	2.9% (2)	1.3% (1)
**Nirmatrelvir + ritonavir**	7.1% (5)	64.3% (45)	18.6% (13)	10% (7)	0% (0)
**Molnupiravir**	7.1% (5)	65.7% (46)	17.1% (12)	10% (7)	0% (0)
**Monoclonals**					
**Casirivimab/imdevimab**	11.4% (8)	65.7% (46)	17.1% (12)	4.3% (3)	1.4% (1)
**Regdanvimab**	0% (0)	72.3% (51)	21.4% (15)	4.3% (3)	1.4% (1)
**Sotrovimab**	25.7% (18)	50% (35)	22.3% (16)	1.4% (1)	0% (0)
**Tixagevimab/cilgavimab**	4.3% (3)	67.1% (47)	27.1% (19)	1.4% (1)	0% (0)

#### Medication use and doses

Steroids were used by 75.6% of respondents (53/70) for COVID-19-specific treatment. Among antivirals, remdesivir was prescribed by 48.6% of respondents (34/70 people). Monoclonals were prescribed by 27.1% (19/70) of practitioners. In 21.4% (15/70) of cases, they were not prescribed because of lack of availability.

The used doses for the listed medications were derived from local hospital protocols. Doses for dexamethasone corresponded to the recommended pediatric dosing. For antivirals and monoclonals, for all patients with a body weight over 40 kg, the standard dosage expected for the adult patient was used. For patients weighing less than 40 kg, dose adjustment formulas were applied according to the literature and international guidelines [14, 15].

The most frequently prescribed medicine among steroids was dexamethasone, at a dose of 0.15 mg/kg/day intravenously for patients of all ages, the youngest aged < 28 days.

Among antivirals, remdesivir was prescribed by most respondents (34/70, 48.6%) at 5 mg/kg intravenously on day one, then at 2.5 mg/kg on days 2–5, even in neonates.

For monoclonals, sotrovimab was prescribed to children as young as two months at 125 mg for patients < 20 kg, 250 mg for those weighing 20–40 kg, and 500 mg over 40 kg.

Further details on less prescribed molecules may be found in Supplementary materials.

#### Clinical benefits

According to 85% of respondents (45/53), steroid therapy had clinical benefits, defined as symptom resolution and/or non-progression to severe disease. Regarding antivirals, 22/32 (68.7%) practitioners observed benefits from remdesivir administration. For monoclonals, observed benefits were reported in more than 50% of the answers.

#### Medicine choice

The choice of monoclonal antibodies was based on the SARS-CoV-2 circulating variant, according to 16/39 answers (41%), and depended on the availability of the medicine at the hospital, according to 11/39 (38.2%) answers.

#### Reported side effects

According to 48/51 (94.1%) respondents, there were no reported side effects related to the administration of steroids. The administration of remdesivir led to side effects according to 7/31 (22.5%) answers. Three respondents reported respectively hypertransaminasemia, renal toxicity, and arrhythmia. According to 42.8% of respondents (3/7), the administration of nirmatrelvir + ritonavir led to hypertriglyceridemia, nausea and vomiting, increased CPK levels, increased levels of concomitant sirolimus, and headache, respectively. No reports of side effects emerged for molnupiravir. Among monoclonals, 2/6 (33.3%) respondents reported headache with vomiting or hypotension and fever at the end of infusion for casirivimab/imdevimab. No adverse effects following administration were reported for sotrovimab and tixagevimab/cilgavimab.

### MIS-C treatment

Detailed data on the use of medications for MIS-C is reported in Table 2.

**Table 2:** Use of medications for MIS-C

	***Yes*, % (*n*)**	***No*, % (*n*)**	***Not available*, % (*n*)**
***Steroids***	79.1% (53)	17.9% (12)	3% (2)
***IVIGS***	69.6% (48)	30% (20)	1.5% (1)
***ASA***	48.5% (32)	50% (33)	1.5% (1)
***Anakinra***	27.9% (19)	70.6% (48)	1.5% (1)

*IVIGs* intravenous immunoglobulins, *ASA* acetylsalicylic acid

#### Medication use and doses

The majority of the respondents (79.1%, 53/67) prescribed steroids for the treatment of MIS-C. However, a high percentage (69.6%, 48/69) also indicated administering IVIGs. A similar number of respondents indicated that steroids and IVIGs were administered simultaneously or in a stepwise manner (21/58, 36.2% and 17/58, 29.3%, respectively).

Both steroids (mostly methylprednisolone at a dose of 2–10 mg/kg/day or dexamethasone 0.2–2 mg/kg/day) and IVIGs (2 g/kg/day) were prescribed to children as young as neonates (< 28 days). ASA (3–5 mg/kg/day) and anakinra (5 mg/kg/day) were administered to patients as young as one year of age.

#### Clinical benefits

According to 90.9% (50/55) of respondents, the administration of steroids led to observed clinical benefits in MIS-C. Similarly, 90.9% (40/44) of respondents described clinical benefits associated with IVIGs. For ASA and anakinra, observed benefits were described by 58.3% (14/24) and 92.3% (12/13) of the respondents, respectively.

#### Reported side effects

For steroid use, 23.9% (11/47) of respondents described bradycardia, hyperglycemia, hypertension, sweating, weight increase, and insomnia. Headache, fluid overload, cardiac failure, and allergic reactions were cited among adverse events after the use of IVIGs, as observed by 30.5% of respondents (11/36). No adverse events following ASA administration were reported in 91.3% of answers (21/23) and anakinra in 80% (12/15).

Free text comments results are included in the Supplementary materials.

## Discussion

This survey represents important information regarding off-label use of COVID-19 and MIS-C therapeutics in children. According to our results, systemic steroids were prescribed in nearly 80% of cases of COVID-19, with almost unanimously perceived clinical benefit and no side effects; however, several molecules and dosing regimens were used. In most cases, dexamethasone was the drug of choice, as it is licensed for use in children and is widely accessible and safe.

Among antivirals, remdesivir was the most frequently prescribed antiviral across all ages, with a good safety profile. Reasons for its widespread use include early authorization by USFDA and its IV administration route, which resulted in higher acceptability, particularly in very young children, compared to oral formulations. Also, there were reports on the compassionate use of remdesivir in children with severe and non-severe COVID-19, with observed clinical benefit and good safety profile [9, 16].

There is already considerable experience with using monoclonals in infancy for RSV [17]. However, the use of monoclonal antibodies in children was inconsistent across countries, and the choice was mainly determined by the SARS-CoV-2 circulating variant or available molecules. According to our survey, monoclonals presented an excellent safety profile in infants and older children. The use in low- and middle-income countries (LMICs) is limited due to costs [18]. More recently, case series and cohort studies in patients younger than 18 years have been published, providing evidence for the good tolerability, with no or rare infusion-related effects of monoclonal antibodies in pediatric age [19–21].

Other recently published retrospective cohort studies reviewed the use of antivirals and monoclonals for early COVID-19 treatment of fragile children at a risk of progression to severe disease, which were well-tolerated [22, 23]. Last, a recent RCT by Upadhyaya et al. provided pharmacokinetic, efficacy, and safety data for the pediatric use of bamlanivimab and etesevimab (BAM + ETE). The drug exposure for weight-based dosing was comparable to the existent data for adults, and the adverse effects were mostly mild or moderate, and consistent with adult data [13].

Survey results also indicate that the mainstay treatments for MIS-C across different countries are steroids and IVIGs, with concurrent administration and stepwise administration (first IVIGs, then steroids) being equally implemented by clinicians. The answers were generally in line with the latest recommendations on the immunomodulatory treatment in MIS-C by the American College of Rheumatology [24], though the choice of the steroid molecule and dosing was not univocal. Clinical benefits were almost universally recognized and the administration of these drugs was generally safe. At the time of the survey no RCTs directly comparing therapeutic approaches in MIS-C had been published, so recommendations were derived from clinical practice and non-randomized comparative cohort studies [25–28]. An RCT has been recently published, aimed at assessing the effectiveness of intravenous methylprednisolone compared with IVIGs for MIS-C treatment, showing that methylprednisolone use did not significantly affect the length of hospital stay as compared to IVIGs, and thus representing an acceptable first-line treatment.

Even though we are now more than two years into the pandemic, the products listed in our survey were not equally available worldwide, especially antivirals other than remdesivir and most monoclonal antibodies, which were mostly missing in hospital facilities in African and South-American countries, Eastern Europe, and Southeast Asia. Some monoclonal antibodies were notably neither available in some facilities the UK and USA. The development of pediatric formulations should be promoted for global access, in line with the WHO Global Accelerator for Paediatric formulations (GAP-f) objectives [11]. The application of this framework to paediatric COVID-19 medicines is therefore crucial also for future pandemics, to allow for rapid clinical assessment, development and registration of age-appropriate formulations and to ensure widespread access to safe and effective drugs, including in LMICs.

### Supplementary Information

Below is the link to the electronic supplementary material.

Supplementary file1 (PDF 1681 KB)

## Data Availability

The datasets generated during and/or analysed during the current study are available from the corresponding author on reasonable request.
