# Supplementary material for: COVID-19 and MIS-C treatment in children—results from an international survey
Source: Eur J Pediatr. 2023 Sep 6;182(11):5087–93. doi: 10.1007/s00431-023-05179-7 (PMC10640428; doi:10.1007/s00431-023-05179-7)
Supplement: Supplementary file 1 — Supplementary file1 (PDF 1681 KB) [file 431_2023_5179_MOESM1_ESM.pdf]

Supplementary Materials

The online questionnaire is available as a PDF document, attached to the *Supplementary Materials*.

Countries of origin of respondents

Among all respondents, 20.5% (15/73) were from Italy, 9.5% (7/73) from the UK, 8.2% (6/73) from Thailand and Spain, 6.8% (5/73) from South Africa and Tanzania, 4.1% (3/73) from Canada and Germany, 2.7% (2/73) from Ethiopia, Pakistan and the Netherlands, 1.3% (1/73) from Bulgaria, Brazil, Poland, Australia, USA, Colombia, Argentina, Lesotho, Peru, Finland, Switzerland, Zambia, France, Uganda, Ukraine, Portugal, Rwanda each.

Global availability by medicine

Remdesivir: Unavailable in some centers in Tanzania, South Africa, Colombia and Lesotho.

Nirmatrelvir + ritonavir: Unavailable in Colombia, Poland, Ukraine, South Africa, Bulgaria, Lesotho, Brazil and Tanzania.

Molnupinavir: Unavailable in Colombia, South-Africa, Bulgaria, Pakistan, Lesotho, Brazil, Tanzania.

Casirivimab-imdevimab: Unavailable in casirivimab-imdevimab was unavailable in centers in Colombia, South Africa, Thailand, Lesotho, Brazil, Tanzania, USA (Texas).

Sotrovimab: Unavailable in Colombia, South Africa, Thailand, Bulgaria, Pakistan, Lesotho, Brazil, UK, Tanzania, Uganda, USA (Texas).

Tixagevimab-cilgavimab: Unavailable in Colombia, South-Africa, Thailand, Bulgaria, Pakistan, UK, Lesotho, Brazil, Uganda, USA (Texas).

COVID-19 treatment – dosing details of other molecules

The youngest patient receiving nirmatrelvir + ritonavir was one year old; the most frequently prescribed dose was 200 mg (30 kg) or 400 mg (adult dose) twice daily (5/70 answers). The youngest reported patient to receive molnupiravir was ten, at a dose of 800 mg twice daily (adult dose, 5/70 answers).

For monoclonals, the administration of casirivimab/imdevimab was reported for children as young as six months of age at variable dosages (8/70 answers).

Children as young as two years of age received tixagevimab/cilgavimab at a dose of 150 mg under 40 kg and 300 mg over 40 kg.

COVID-19 treatment - Reasons behind medicine choice

The choice of monoclonal antibodies was based on the SARS-CoV-2 circulating variant, according to 16/39 answers (41%), and depended on the availability of the medicine at the hospital, according to 11/39 (38.2%) answers. Other reasons guiding the medicine of choice, mentioned by at least one respondent, included the patient's age, immunosuppression state, the prolonged activity of the drug, risk stratification according to disease severity and national policy, clinical trial access and risk/benefit ratio for the individual patient, including serostatus and variant.

Free-text comments

Seven respondents from the UK, South Africa and Germany mentioned using tocilizumab in the final free-text comments. Tocilizumab use was described for children and young adolescents (excluding infants aged below one year) COVID-19 and MIS-C alike, mainly in the context of the RECOVERY trial, often as a rescue treatment for COVID-19 cases. Respondents indicated that tocilizumab was administered intravenously (IV) at a dose of 12 mg/kg (single dose) and 8 mg/kg (maximum 800 mg) for people aged under 12 years (<30 kg) and above 12 years (>30 kg),

respectively. Respondents indicated that it was challenging to state the benefits of drug administration; one responder reported rapid improvement in an 8-year-old patient.

Covid-19 and MIS-C management and treatment survey

* Indica una domanda obbligatoria

1. Email *

2. **Country** *

3. **Center name**

Management of Covid-19 in pediatric patients - general management

4. **Did you use steroids for the clinical management of pediatric Covid-19 patients?** *

Contrassegna solo un ovale.

- yes
- no
- not available
- No, available but not approved for children with COVID-19
- No, available but not approved for COVID-19
- Altro: ______

5. **How many patients were treated with steroids for Covid-19?**

6. **How old was the youngest patient you prescribed steroids to?**

7. **Please, specify drug name and dose (mg/kg/day)**

8. **Did you observe any clinical benefit as a result?**

9. **Did you report any side effect in relation to the drug being administered?**

Management of Covid-19 in pediatric patients - Antivirals

10. **Did you use Remdesivir for the clinical management of pediatric Covid-19 patients?** *

Contrassegna solo un ovale.

- yes
- no
- not available
- no, available but not approved for children with COVID-19
- no, available but not approved for COVID-19
- Altro: ______

11. **How many patients were treated with Remdesivir for Covid-19?**

12. **How old was the younger patient you prescribed Remdesivir to?**

13. **Please, specify dose (mg/kg/day)**

____

14. **Did you observe any clinical benefit as a result?**

____

15. **Did you report any side effect in relation to the drug being administered?**

____

16. **Did you use Paxlovid (PF-07321332/ritonavir) for the clinical management of pediatric Covid-19 patients?** *

Contrassegna solo un ovale.

- yes
- no
- not available
- no, available but not approved for children with COVID-19
- no, available but not approved for COVID-19
- Altro: _______________________

17. **How many patients were treated with Paxlovid (PF-07321332/ritonavir) for Covid-19 ?**

____

18. **How old was the youngest patient you prescribed Paxlovid (PF-07321332/ritonavir) to?**

____

19. **Please, specify dose (mg/kg/day)**

____

20. **Did you observe any clinical benefit as a result?**

____

21. **Did you report any side effect in relation to the drug being administered?**

____

22. **Did you use Lagevrio (molnupiravir for the clinical management of pediatric Covid-19 patients?** *

Contrassegna solo un ovale.

- yes
- no
- not available
- no, available but not approved for children with COVID-19
- no, available but not approved for COVID-19
- Altro: _______________________

23. **How many patients were treated with Lagevrio (molnupiravir) for Covid-19?**

____

24. **How old was the youngest patient you prescribed Lagevrio (molnupiravir) to?**

____

25. **Please, specify dose (mg/kg/day)**

____

26. **Did you observe any clinical benefit as a result?**

____

27. **Did you report any side effect in relation to the drug being administered?**

____

Management of Covid-19 in pediatric patients - Monoclonal antibodies

28. **Did you use Ronapreve (casirivimab-imdevimab) for the clinical management of pediatric Covid-19 patients?** *

Contrassegna solo un ovale.

- yes
- no
- not available
- no, available but not approved for children with COVID-19
- no, available but not approved for COVID-19
- Altro: ______________________________

29. **How many patients were treated with Ronapreve (casirivimab-imdevimab) for Covid-19?**

____

30. **How old was the youngest patient you prescribed Ronapreve (casirivimab-imdevimab) to?**

____

31. **Please, specify dose (mg/kg/day)**

____

32. **Did you observe any clinical benefit as a result?**

____

33. **Did you report any side effect in relation to the drug being administered?**

____

34. **Did you use Regkirona (regdanvimab) for the clinical management of pediatric Covid-19 patients?** *

Contrassegna solo un ovale.

- yes
- no
- not available
- no, available but not approved for children with COVID-19
- no, available but not approved for COVID-19
- Altro: _______________

35. **How many patients were treated with Regkirona (regdanvimab) for Covid-19?**

____

36. **How old was the youngest patient you prescribed Regkirona (regdanvimab) to?**

____

37. **Please, specify dose (mg/kg/day)**

38. **Did you observe any clinical benefit as a result?**

39. **Did you report any side effect in relation to the drug being administered?**

40. **Did you use Xevudy (sotrovimab) for the clinical management of pediatric Covid-19 patients?** *

Contrassegna solo un ovale.

- yes
- no
- not available
- no, available but not approved for children with COVID-19
- no, available but not approved for COVID-19
- Altro: ____________

41. **How many patients were treated with Xevudy (sotrovimab) for Covid-19??**

42. **How old was the youngest patient you prescribed Xevudy (sotrovimab) to?**

43. **Please, specify dose (mg/kg/day)**

44. **Did you observe any clinical benefit as a result?**

____

45. **Did you report any side effect in relation to the drug being administered?**

____

46. **Did you use Evusheld (tixagevimab-cilgavimab) for the clinical management of pediatric Covid-19 patients?** *

Contrassegna solo un ovale.

- ( ) yes
- ( ) no
- ( ) not available
- ( ) no, available but not approved for children with COVID-19
- ( ) no, available but not approved for COVID-19
- ( ) Altro: _______________

47. **How many patients were treated with Evusheld (tixagevimab-cilgavimab) for Covid19?**

____

48. **How old was the youngest patient you prescribed Evusheld (tixagevimab-cilgavimab) to?**

____

49. **Please, specify dose (mg/kg/day)**

____

50. **Did you observe any clinical benefit as a result?**

____

51. **Did you report any side effect in relation to the drug being administered?**

____

52. **Please state if the choice of monoclonal antibody was determined by**

Contrassegna solo un ovale.

- ( ) the SARS-CoV-2 circulating variant at that moment
- ( ) the available molecule at the Hospital
- ( ) Altro: _______________________

MIS-C management and treatment

53. **Did you use steroids for the clinical management of MIS-C?** *

Contrassegna solo un ovale.

- ( ) yes
- ( ) no
- ( ) Altro: _______________________

54. **How many patients were treated with steroids for MIS-C?**

____

55. **How old was the youngest patient you prescribed steroids to?**

____

56. **Please, specify drug name and dose (mg/kg/day)**

____

57. **Did you observe any clinical benefit as a result?**

____

58. **Did you report any side effect in relation to the drug being administered?**

____

59. **Did you use Immunoglobulins (IVIGs) for the clinical management of MIS-C?** *

Contrassegna solo un ovale.

- yes
- no
- Altro: _______________________

60. **How many patients were treated with IVIGs for MIS-C?**

____

61. **How old was the youngest patient you prescribed IVIGs to?**

____

62. **Please, specify drug name and dose (mg/kg/day)**

____

63. **Did you observe any clinical benefit as a result?**

____

64. **Did you report any side effect in relation to the drug being administered?**

65. **Please, specify if**

Contrassegna solo un ovale.

- both steroids AND IVIGs were always administered
- IVIGs and steroids were administered in a stepwise process (first IVIGs, then steroids)
- Altro:

66. **Did you use ASA for the clinical management of MIS-C?** *

Contrassegna solo un ovale.

- yes
- no
- Altro:

67. **How many patients were treated with ASA for MIS-C?**

68. **How old was the youngest patient you prescribed ASA to?**

69. **Please, specify dose (mg/kg/day)**

70. **Did you observe any clinical benefit as a result?**

71. **Did you report any side effect in relation to the drug being administered?**

72. **Did you use Anakinra for the clinical management of MIS-C?** *

Contrassegna solo un ovale.

- yes
- no
- Altro:

73. **How many patients were treated with Anakinra for MIS-C?**

74. **How old was the youngest patient you prescribed Anakinra to?**

75. **Please, specify dose (mg/kg/day)**

76. **Did you observe any clinical benefit as a result?**

77. **Did you report any side effect in relation to the drug being administered?**

78. **Did you use ACE-i for the clinical management of MIS-C?** *

Contrassegna solo un ovale.

- yes
- no
- Altro: ____________

79. **How many patients were treated with ACE-i for MIS-C?**

____

80. **How old was the youngest patient you prescribed ACE-i to?**

____

81. **Please, pecify drug name and dose (mg/kg/day)**

____

82. **Did you observe any clinical benefit as a result?**

____

83. **Did you report any side effect in relation to the drug being administered?**

____

Off-label use in Covid-19 and/or MIS-C treatment

84. **How many and which molecules did you use off-label in children, for treatment of Covid-19 and/or MIS-C?**

Comments

85. **Please, add any comments**

Questi contenuti non sono creati né avallati da Google.

Google Moduli
